# The collaboration of general practitioners and nurses in primary care: a comparative analysis of concepts and practices in Slovenia and Spain

**DOI:** 10.1017/S1463423617000354

**Published:** 2017-06-20

**Authors:** Kerstin Hämel, Carina Vössing

**Affiliations:** 1 Professor of Public Health, Health Services Research in Nursing, School of Public Health, Bielefeld University, Germany; 2 Research Associate, Working Group Health Services Research & Nursing Science, School of Public Health, Bielefeld University, Germany

**Keywords:** cross-professional, nursing roles, primary health care, Slovenia, Spain, team-based

## Abstract

**Aim:**

A comparative analysis of concepts and practices of GP-nurse collaborations in primary health centres in Slovenia and Spain.

**Background:**

Cross-professional collaboration is considered a key element for providing high-quality comprehensive care by combining the expertise of various professions. In many countries, nurses are also being given new and more extensive responsibilities. Implemented concepts of collaborative care need to be analysed within the context of care concepts, organisational structures, and effective collaboration.

**Methods:**

Background review of primary care concepts (literature analysis, expert interviews), and evaluation of collaboration in ‘best practice’ health centres in certain regions of Slovenia and Spain. Qualitative content analysis of expert interviews, presentations, observations, and group discussions with professionals and health centre managers.

**Findings:**

In Slovenian health centres, the collaboration between GPs and nurses has been strongly shaped by their organisation in separate care units and predominantly case-oriented functions. Conventional power structures between professions hinder effective collaboration. The introduction of a new cross-professional primary care concept has integrated advanced practice nurses into general practice. Conventional hierarchies still exist, but a shared vision of preventive care is gradually strengthening attitudes towards team-oriented care. Formal regulations or incentives for teamwork have yet to be implemented. In Spain, health centres were established along with a team-based care concept that encompasses close physician–nurse collaboration and an autonomous role for nurses in the care process. Nurses collaborate with GPs on more equal terms with conflicts centring on professional disagreements. Team development structures and financial incentives for team achievements have been implemented, encouraging teams to generate their own strategies to improve teamwork.

**Conclusion:**

Clearly defined structures, shared visions of care and team development are important for implementing and maintaining a good collaboration. Central prerequisites are advanced nursing education and greater acceptance of advanced nursing practice.

## Background

Current discussions in health studies concerning the need for high-quality health care are focussed on cross-professional^1^ teamwork as a key issue in improving health care quality (cf. Thylefors *et al*., 2005; Samuelson *et al*., 2012). The view that cross-professional collaboration is necessary for high-quality care is, however, not new; as early as 1978, the International Conference on Primary Health Care (Alma-Ata) pronounced cross-professional health teams as essential to meeting the multiple (primary) health needs within the community (WHO, 1978). Since then, many countries have followed this vision, implementing primary care teams as well as multi-professional primary health centres and thus providing comprehensive care by integrating health promotion activities, preventive, curative, and rehabilitative care (Saltman *et al*., 2006; WHO, 2008; Hämel and Schaeffer, 2014). In the light of today’s fast-ageing population and the increasing number of patients with complex needs, the potential for cross-professional collaboration in the field of primary health care has become even greater than the founders of the Alma-Ata Declaration and initiators of health teams originally imagined. The World Health Report 2008 (46f) indicated that primary care teams are better able to optimise the care process by assuming the coordinator role, thus avoiding task fragmentation and improving the continuity of care even for high-demand patients.

Primary health care can actually be seen as a key impetus for innovation in cross-professional collaboration. Working in cross-professional health teams has challenged care concepts dominated by physicians and led to the implementation of new and more extensive responsibilities for nurses, midwives, physiotherapists, psychologists, social workers and other health professionals working alongside family physicians to promote patient-centred and community-oriented care (Kendall, 2008; Freund *et al*., 2015: 738; Kringos *et al*., 2015: 31). Thus, team-based primary health care has led to a greater professionalisation of nurses and other non-medical health professionals, freeing them from their customary subordinate role in health care. Initial research shows that particularly nurses have benefited from this recent development. New chronic care concepts implemented in many countries in the past few years have strengthened nurses’ role as a first contact partner in primary health endeavouring to establish long-standing nurse–patient relationships (cf. Schaeffer *et al*., 2015; Kendall and Bryar, 2017). Consequently, this requires a close collaboration between the personal GP and the personal nurse (cf. Hämel and Schaeffer, 2014).

Optimally, each health professional in a cross-professional team is responsible for his or her specialised field. Combining the skills, experience and expertise of each profession in a team is seen as the main benefit: the team members have access to diverse knowledge and competencies; this enables the team to provide a broad spectrum of services and generate a more holistic view of the patients’ situation (Thylefors *et al*., 2005: 102–103). Collaboration between the professionals helps prevent shortcomings of sequential care processes, facilitates learning from other disciplines and increases patient satisfaction as well as job satisfaction (Thylefors *et al*., 2005; Wen and Schulmann, 2014; Morgan *et al*., 2015).

Although criticised for certain methodological shortcomings, some studies investigating the effects of team-based care have provided evidence of positive patient outcomes such as higher self-perceived health and life satisfaction as well as higher satisfaction with health care (Martin *et al*., 2010; Schepman *et al*., 2015). Other studies denote cost-effectiveness of team-based approaches in primary care (Jacobson and HDR Inc., 2012; Mundt *et al*., 2015). Even so, research findings regarding effectiveness and outcomes of cross-professional collaboration, in general, remain ambiguous (Barrett *et al*., 2007).

The most common obstacles to collaborating work in cross-professional teams evolve around organisational and professional separatism (Jamieson and Illsey, 1989; Frenk *et al*., 2010). Researchers have established that for effective teamwork between GPs and nurses collaboration needs to be based on an understanding of each other’s professional identity and specific role in the care process as well as on mutual respect and trust (Pullon, 2008; Jaruseviciene *et al.*, 2013). Conventional hierarchical structures between health professionals hamper collaboration through differences in status and sustained professional rivalry (Nancarrow *et al*., 2013; Schadewaldt *et al*., 2013; Supper *et al*., 2015; Schaeffer and Hämel, 2017). The prospect of working (more) independently of the physician is a key impetus for effective collaboration on equal footing. However, this is not a matter of course, not even for the well-educated and highly trained nurse practitioners (Schadewaldt *et al*., 2013).

In addition, research has confirmed that co-location and a ‘robust organisation’ with formal supportive structures for team-oriented care facilitate effective teamwork (Oandasan *et al*., 2009; Munro *et al*., 2013; Schaeffer and Hämel, 2017). Advantageous are also shared goals, good leadership, clear task division between health professionals, strong communication and regular appraisal of team success (Bodenheimer, 2007: 6; Xyrichis and Lowton, 2008: 149–150; Kennedy *et al*., 2015: 362). Consequently, health systems with fragmented health care services can be particularly challenging to cross-professional collaboration: a long-established organisational separation of primary care services – health, social, preventive and curative care – promotes incompatible objectives and interests of the involved organisations (Delamaire and Lafortune, 2010; Schaeffer and Hämel, 2017).

As primary health care has had to adapt to changing patient needs, professional tasks and roles have changed, for example, professionals now have to support patients in their self-management of chronic conditions. In the last years, many countries have also encouraged a shift in responsibilities for certain tasks from physicians to nurses, in particular (but also to other health professionals) (Maier and Aiken, 2016). Both the new responsibilities in patient care and the task shifting from physicians to nurses necessitate a redesign in primary health care teams and, consequently, a restructuring of cross-professional collaboration.

Given that many countries are faced with these challenges today, it appears prudent to investigate and compare the different concepts being implemented and the experiences of those involved. This paper, therefore, examines the concepts and experiences of the cross-professional collaboration of GPs and nurses found in Slovenian and Spanish primary health centres. While bearing the specific health care context and traditions in mind, we will focus here on the effectiveness of collaboration.

Slovenia and Spain were chosen for this comparison, because they both have robust primary health care systems (Kringos *et al*., 2015) and have operated cross-professional centres for many years now. It can hence be assumed that physicians and nurses are well experienced in collaboration (Albreht *et al.*, 2009; Dedeu *et al*., 2015). In addition, many Slovenian and Spanish primary health centres have increased their efforts as of late to strengthen teamwork between GPs and nurses in their primary health centres. The following paper will begin with a short sketch of each country’s primary care concept. That will be followed by a closer examination of their approaches to teamwork and recent developments. The prospects for cross-professional collaboration between GPs and nurses will then be compared and discussed.

## Method

The findings presented in this paper are focused on concepts and practices of cross-professional collaboration of GPs and nurses in Slovenian and Spanish health centres. They draw on a narrative review of literature available in English on the development of cross-professional primary health care in Slovenia and Spain combined with a qualitative analysis of the current situation in selected health centres based on semi-structured expert interviews, presentations, group discussions and recorded observations.^2^

The literature used for the analysis included academic studies published in Slovenia and Spain as well as internationally. These publications focused on the development of primary care and primary care concepts in the two countries as a whole, in addition to primary care teamwork, cross-professional collaboration as well as the role and position of GPs and nurses in the teams in particular. Government documents, official statistics and national reports provided information about the health systems in general, on planned and initiated reforms and basic data on primary care. Interviewed experts in both countries also recommended relevant literature on primary care and on the role of the health professions and primary care providers in each country. Furthermore, they supported us in identifying interesting concepts of cross-professional collaboration.

A major challenge of this study was to gain practical access to health care centres in the chosen countries. In a first step, key experts on primary health care in both countries were identified with help of the literature analysis and through contacts in the European Network for Primary Care. They were then contacted with the request for an interview. Ten expert interviews^3^ relating to Spain and Slovenia were conducted (see Supplementary material, Table A1). The semi-structured interviews focused on past and recent developments in primary care and cross-professional collaboration in the different countries. Key characteristics and innovative elements of health care centres, roles and tasks assumed by the various health professionals were of particular interest as well as the interaction and co-operation between team members.

The interviewees were also asked to recommend health care centres in the country of their expertise, which they consider particularly good examples of cross-professional teamwork and collaboration in primary health centres. The recommended centres were then contacted with a request to visit. The participants were informed beforehand of the study’s objectives and consented to the audio recording of the entire visit. A weeklong trip was scheduled for each country. In total, seven health centres (Slovenia: three, Spain: four) and their associated health stations were visited. In Slovenia, the primary health centres visited were spread across the country. Due to Spain’s geographical size and time restrictions, visitations there were limited to health centres in Catalonia and the Basque Country – both regions were chosen based on experts’ recommendations.

The visits to each health centre were organised similarly, though they differed in detail. Most began with a presentation by the health centre managers summarising the health centre’s vision, purpose and the organisational structure. This was followed up by a guided tour of the complex. The authors then jointly conducted a series of semi-structured interviews with various health professionals. The interviews focused mainly on key tasks and responsibilities, co-operation in the team and with associated partners of the health centres, for example, secondary care, social services. Additional interviews were conducted with the health centre’s managers focusing on key characteristic and innovative aspects, development, implementation and evaluation of the care concepts, the daily routine, roles in the team and services provided. Finally, in some cases, the centre’s management organised group discussions with health professionals to delve deeper into specific aspects. Since these group discussions were organised spontaneously on site by the hosts, it was not possible to prepare methodical guidelines for them beforehand. They were nevertheless crucial for the understanding of the given health care concepts. The authors were able to ask extensive questions, voice observations and tentative assessments on the care concepts and cross-professional collaboration of the health centre, and discuss these in-depth with the participants. All presentations, interviews, group discussions and observations during the tours through the health centres were digitally recorded.^4^ Practically all interviews, presentations and tours were conducted in English as a second language for all participants; only one interview was conducted in German. Where necessary, the interviewees were assisted by a translator.

The recorded material was transcribed as needed: the interviews for the most part as full scripts, the presentations and centre tours as summaries, the group discussions as a combination of script and summary. The data was subsequently analysed by means of computer-assisted qualitative content analysis using MAXQDA (cf. Meuser and Nagel, 1991; Gläser and Laudel, 2004). The individual transcripts were first roughly structured according to the topics discussed in the interviews. The material was then re-read, and inductive categories were defined (eg, status of the nurses). The entire text material was encoded, and individual sections of the text were analysed and interpreted in more detail. The authors worked jointly in all phases of the data collection and content analysis.

A case description of each country was then composed identifying the influencing historical and cultural factors, current cross-professional team model as well as key structures of the model. Experts in the field were given short presentations of our initial findings and research hypotheses. The ensuing discussions added valuable insights to the analysis. Finally, the case descriptions were used as a basis for the iterative, discursive process that followed, in which the authors sought to identify key components of team concepts used in each country, their similarities as well as differences. The triangulation of various data sources, multiple observers and the integration of the varied perspectives of the researchers as well as external experts in the course of the analysis allowed us to cross-check our findings and provided us with a deeper understanding of the concepts and practices of cross-professional primary care in Slovenia and Spain.

## The case of Slovenia

In Slovenia, the first implementation of multi-professional primary health centres took place in the 1920s. The aim was to provide general medical care, mother, and childcare and preventive medical care for communicable diseases (such as tuberculosis) for vulnerable groups in rural areas (Albreht *et al*., 2006: 238). After the Second World War, Yugoslavia’s national health policy strategy was to build up the health care system around primary health care centres (Saric and Rodvin, 1993; Klančar and Svab, 2014: 167). Accordingly, the many health centres built in the 1950s and 1960s were responsible for the health needs of the population in specified geographical areas (Albreht *et al*., 2006: 238; Klančar and Svab, 2014: 167). To build up an adequately trained workforce for primary care vocational training for community nursing was introduced in 1957 (Slajmer-Japelj, 1993: 328; Hennessy and Gladin, 2006: 40) and special training for GPs in 1962 (Bulc *et al*., 2006).^5^ After their declaration of independence and the founding of the state Slovenia in 1991, a national health insurance system was initiated (Albreht *et al*., 2016: 21). Under political pressure at the time to ‘free’ the professionals, the government allowed health professionals to open private practices, also hoping to provide thus a more efficient patient-friendly health care (Albreht and Klazinga, 2009: 82).^6^ Even so, the health centres owned by the municipalities continued to play a central role in primary health care with 76.5% of the primary health care physicians working in municipal health centres. Most recent statistics (2013) count 65 health centres represented in 459 locations that cover all regions of Slovenia (Albreht *et al*., 2016: 118).

Today, the Slovenian health centres offer a broad spectrum of services: Family medicine, health care for women, children and youths, community nursing, physiotherapy, occupational health, laboratory and other diagnostic services, dental care and emergency care (Albreht *et al*., 2016: 130). Diverse professionals must collaborate to provide such comprehensive services: family physicians, paediatricians, gynaecologists, registered nurses,^7^ nurse assistants, midwives, physiotherapists, speech therapists, occupational therapists, dentists, psychologists/psychiatrists and other health professionals (Rotar Pavlič *et al*., 2015: 246; Albreht *et al.,* 2016: 119).^8^ The GPs and the community nurses^9^ are the patients’ points of first contact (Int-Sp25, 606). Both are organised in separate independent units. Since 2011, ‘practice nurses’ also provide first contact (Albreht *et al*., 2016: 111). Before taking a closer look at this new development, we will outline the given forms of collaboration between physicians and community nurses.

### Collaboration between GPs and community nurses

In a general practice unit in Slovenia, each GP organises his or her own patient list with the administrative support of an assistant nurse. Community nurses are, in contrast, responsible for the population in a specified geographical area (~2500 persons) as a whole. They plan their daily routines in the communal nursing unit. They make house calls for pregnant women, newborn and schoolchildren monitoring their health status, detecting health problems and answering questions about pregnancy and childcare (Int-Slo1, 153–158). Increasingly now, community nurses are also providing preventive care during home visits for older people^10^ assessing the health and social situation, monitoring health parameters, providing health counselling and health education (Int-Slo3, 104–111). Both areas of work are defined as ‘preventive care tasks’ and distinguished from nurses’ ‘curative tasks’ (Int-Slo5, 117). Community nurses work independently of the GPs when dealing with preventive care tasks; they plan and coordinate their activities autonomously (Int-Slo5, 118). Their responsibilities are, however, limited once health problems are manifested. In such cases, the patient is advised to see the physician. Nurses follow physician’s orders regarding the home treatment when executing their curative tasks. The GP determines the care plan for patients, and nurses report to the GPs concerning changes in health status (Int-Slo1, 302–307). Due to their expertise and knowledge gained from the patients’ situation, the familial and social context over the many years of attendance, the Slovenian community nurses interviewed felt capable (to a certain degree) of assessing patients’ situation and adjusting care plans.

### Collaboration on demand

The interviewees claim that nurses and physicians in Slovenia collaborate well. They are able to discuss questions regarding the care plan or complications. However, *collaboration appears to be driven by the nurses’ interests* and shaped by their limited scope of practice. The perceived quality of collaboration is dependent on personalities, personal inclinations and expectations.

‘Informally, we work quite well together. I call the general practitioner and say “this patient needs to be visited at least every second day, but you wrote [down] only once a week; he [the patient] has this, this and this”. If I explain [the situation] using some [good] arguments, it is not a problem’.^11^ (Int-SloE3, 160–162, Community nurse)

‘Mainly, it [collaboration] is over the phone, and we have these kinds of forms so that things are written down: Information for the doctor if there are any changes. Sometimes, we also go to the general practice to talk to him personally, but that’s not very common’. (Int-Slo10, 343-351, Community nurse)

Interestingly enough, the structural separation of the professions in different care units has two sides to it concerning collaboration between GPs and community nurses: On the one hand, the separate organisation of nurses gives them more autonomy to develop their practice further. On the other hand, the restrictive scope of tasks community nurses are allowed to perform, forces the nurses to work closely with the GPs and to a certain extent, be controlled by them. Furthermore, the lack of a homogeneous concept of collaboration in the sense of working together towards a common goal as mutually respected partners hinders efficient collaboration.

### Model practices: new possibilities for collaboration

The implementation of the so-called ‘model practice’ in Slovenia in 2011 represents a step forward towards closer collaboration between GPs and nurses. The model is based on the concept of a GP-nurse tandem with a well-defined plan for the division of responsibility and the integration of a practice nurse in a GP practice. Practice nurses in Slovenia are graduate nurses, specially trained to provide screening and prevention of eight chronic diseases and chronic disease risk factors^12^ for the population 30 years and older (Poplas Susič *et al*., 2015: 636; Albreht *et al*., 2016). In their consultations, these nurses provide health information, education and counselling of patients to promote healthy lifestyles. The practice nurses follow up patients with chronic diseases or high-risk thereof by monitoring their health status and supporting their self-management. The nurses have to follow nationwide implemented protocols that clearly define measures and methods of treatment. These protocols also stipulate when nurses are to consult or refer the patient to a physician (Poplas Susič *et al*., 2015).

The model practice was introduced to strengthen preventive care in the general practice (Albreht *et al*., 2016: 116). Supported by the Ministry of Health as a pilot project (Poplas Susič *et al*., 2015: 162), the model practice was quickly implemented with the set goal that all general practice units adapt the new health model by the end of 2018 (Albreht *et al*., 2016: 162). The transfer of responsibilities from physicians to nurses was discussed *controversially*; however, in the end considering their own heavy workload, it was accepted by most physicians (Poplas Susič *et al*., 2015; Rotar Pavlič *et al*., 2015: 247).

The concept gives Slovenian nurses a new area of practice with a higher level of autonomy. The practice nurses plan their schedules and procedures independently. Interviewed nurses demonstrated a *high level of job satisfaction* underscoring the opportunity to work with diverse patients under varied circumstances (healthy and sick, young and old). They point out, in particular, the advantages of the offered health education and counselling to improve patients’ health and well-being. Moreover, the data collected verify that even in GP-nurse tandems, the ‘team spirit’ evolves from a *shared vision of a more patient-centred, responsive care* that can be provided by these new services.

‘Our medicine has changed; the patient now [comes] in the centre and we have to work together for the benefit of the patient’. (Int-Slo10, F24, medical director)

The model practice has also changed the routines for the GPs, who were used to having sole responsibility for their practice. In keeping with the new model, they are expected to coordinate patients’ care with the practice nurse by team collaboration (ie, exchange knowledge about the patient’s situation und discussing patient’s conditions).

### Reduction of the GP’s workload as impetus and barrier for team-based care

The misuse of the *practice nurses to reduce the GP’s immense workload* has been identified as a major obstacle of teamwork. Occasionally, GPs fall back into their traditional role of the taskmaster exploiting the GP-nurse tandem to delegate supportive tasks to the practice nurse:

‘She’s helping […]. So when I need her, she’s here’. (Int-Slo4, 90-92, GP)

‘We are trying to implement it, but we are not always successful in lightening the burden on the registered nurse’. (Int-Slo3, 187-191, GP)

Although physicians’ considerable workload has often been an excellent incentive for accepting the integration of practice nurses, once the concept has been accepted it also appears to be a central barrier to any further development of inter-professional collaboration.

‘We have difficulties forming a team, where they are partners’. (Int-Slo11, 273-281, GP/Medical director)

In summary: The introduction of the model practice into the Slovenian health system based on a stringent team-based concept has led to improved preventive health care and health consultation that is more responsive to patients needs. Nevertheless, traditional professional hierarchies have yet to be overcome, and they continue to hamper effective implementation. Even though this door-to-door co-operation has achieved a new and higher degree of collaboration, new challenges are rising. Practicing preventive health care closely together as a team means nurses must learn to withstand the traditional dominance of the physicians to develop further and defend their new care role, even when they are currently in the midst of just learning to fulfil it.

## The case of Spain

Multi-professional primary health centres were first established in Spain in the 1980s as a part of the overall democratisation process initiated at the end of the Franco regime. The implementation of health centres was accompanied by a transformation of the health system from a social insurance model to a tax-based national health service (European Observatory – European Observatory on Health Care Systems, 2000). The health centres provide the Spanish population with universal access to health care, integrating preventive, curative and rehabilitative services under one roof (Hart, 1990; Borkan *et al*., 2010: 1433; García-Armesto *et al*., 2010). Cross-professional primary health care teams were set up to take over a shared responsibility for the primary health care of the population in a specific geographical area (García-Armesto *et al*., 2010: 57; López, 2011). As of 2003, primary health care teams are operated in all regions of Spain (Gené-Badia *et al*., 2008: 2). A network of 3023 health centres and 10 081 health stations exists today (MoHSE, 2015: 9). Most of them are public organisations administrated by regional health services (ie, the Catalan Institute of Health).^13^

The cross-professional teams are composed of GPs, pediatricians, nurses, nurse assistants and administrative staff. Depending on regional concepts, they can also include dentists, social workers, midwives and physiotherapists (Borkan *et al*., 2010: 1434; Dedeu *et al*., 2015: 257). The teams presently claim a ratio of nearly 1:1 physicians to nurses (MoHSE, 2015: 10). The health centres offer comprehensive services for adults as well as specialised health care services for older people, women, children and youths (García-Armesto *et al*., 2010: 138). Their services encompass individual care, clinical care at the health centre, home care and community health activities.

Regarding the collaboration between physicians and nurses in Spain, two aspects are of particular interest. First, with the implementation of primary care teams, the nursing profession was given a new status in primary care with advanced responsibilities. An important prerequisite for this role enhancement was the introduction of nursing education as degree level study programmes in 1977 (Mariscal Crespo *et al*., 2010: 341; López, 2011: 1722). Since 1990, the curricula of nursing education focus in particular on community care (Zabalegui Yarnoz, 2002: 313; López, 2011: 1722).^14^ Second, since the health centres were established, inter-professional teamwork has been seen as crucial for effective primary care (Goñi, 1999: 108; Dedeu *et al*., 2015: 255). The professionals have not simply been gathered under one roof. They have worked hard specifically to develop and strengthen teamwork as well. For example, supplement programmes have been initiated, such as joint ventures for research projects, special programmes for team-based community action and health promotion activities (Martí Arguasca and Argimon, 2008; Dedeu *et al*., 2015). Furthermore, in several autonomous regions ‘productivity bonuses’ (eg, for the judicious use of prescriptions or the reduction of waiting time) have been introduced, which in part are rewarded as team bonuses (García-Armesto *et al*., 2010: 116; MoHSE, 2010: 62).

### Doctors for acute cases, nurses for chronic conditions

In a health centre, each patient has a personal GP and a personal nurse, who are his or her first contact point (Contel and Badia, 1998: 42; Dedeu *et al*., 2015: 258). Teamwork of GPs and nurses is based on the concept of shared responsibility: GPs treat primarily the acutely ill and unstable chronic patients; nurses are responsible for chronic patients in stable conditions (López, 2011). Both professionals have their own patient list; however, in most cases, the GP and nurse work together for the same patient base (Int-Sp8, 352).^15^

Within their scope of practice, nurses make decisions independently (Dedeu *et al*., 2015). Compared to the more functional differentiation of nursing roles in Slovenian primary care, primary care nurses in Spain commonly perform a broader range of activities (general nurses). They conduct individual patient consultations at the health centre, home care for immobile patients as well as health promotion activities at the health centre and in community settings^16^ (Martí-Arguasca and Argimon, 2008: 11; Int-Sp7, 66–72; Int-Sp1, 114–127). During the patient consultations, nurses make a comprehensive assessment of the social and health situation of the patient, including lifestyle and health-related behaviour. They also follow-up the health status of patients and give self-management support. The information that nurses collect is of great relevance for the GPs as well; and vice versa, GPs’ diagnosis and treatment plan are essential for nursing care. In Spain, the exchange of information is facilitated using an electronic health records system, which has been established in nearly all Spanish health centres (Borkan *et al*., 2010). This tool allows each professional access to the diagnosis, further developments regarding the patient’s health, current health status and treatment plan.

### Teamwork on equal footing

GPs and nurses set up the care plan as a team (Dedeu *et al*., 2015). In cases of increasing complexity, co-operation is intensified. For example, when dealing with immobile patients with complex health needs, joint visits at patient’s home may be arranged (Int-Sp1, 124).^17^ In the comprehensive field of chronic care (including health promotion and self-management support), nurses are accredited with a distinguished status due to their separate field of expertise. GPs and nurses appear to collaborate closely when coordinating patients’ care. To attain a holistic view of patients’ situation, they have to combine their expertise and care skills. Our analysis shows that this arrangement often works efficiently. In many teams, the physicians and nurses value one another as equals and work together as partners.

‘Nurses and doctors are talking at the same level and integrate their competencies’. (Int-Sp2, 29, Nursing Director)

Nevertheless, collaboration conflicts are inevitable. In some teams, the working environment is less congenial:

‘In some teams, there is a good collaborative atmosphere; in others, there are lots of battles between doctors and nurses’. (Int-SpE5, 140-142, Centre manager)

‘But it’s not the same for everybody. Some doctors are stiffer. Some are [more relaxed], and then they are easier to work with’. (Int-Sp12, 357–358, Nurse)

In other words, conflicts can hinder effective collaboration. Obviously, teamwork is shaped by the personalities of individuals. It is also influenced by the degree of willingness to work together. Specifically to cope with such problems, certain processes and structures have been implemented to facilitate collaboration in Spain.

### Team co-operation and team development

A measure used to promote team collaboration in the Spanish health centres is to include time in their weekly schedule for team activities (Goñi, 1999: 108; Dedeu *et al*., 2015). The frequency of these time slots varies from once a week to daily (Int-Sp21, 80; Int-Sp22, 109). This scheduled time is used for team meetings, training or other joint activities for the entire health centre staff. During the meetings, the team members convene to discuss organisational issues or complex cases, exchange views on new programmes as well as deliberate current qualification requirements (Int-Sp3, 33–35). The joint participation of (inter-)professional training may be planned as well. The health centres also offer team members opportunities for further training and thus support professional advancement in the team. Such opportunities help broaden the spectrum of services provided, but even more important, it motivates the professionals to develop their individual expertise further according to their interests and capabilities (eg, nutrition, emergency care). The idea is that the team as a whole can benefit from the knowledge gained. The trained health professionals become first contacts for questions that arise in the team concerning their field of expertise. Team projects are also encouraged as another way of promoting specialisation; for example, improving established processes or developing new modes of teamwork (Int-Sp3, 113–123; Int-Sp6, 263–267).

Team development also comprises the advancement of professional roles. In Spain, the teams have implemented their own processes locally. Nurses, in particular, have been allowed to take on responsibilities beyond set standards. For example, the authorisation for nurses to prescribe medications is pending legislative approval for many years. As a reaction, several local teams developed their own procedure to enable nurses to prescribe medications when necessary by devising special protocols (Int-Sp1, 291).^18^

Finally, an attempt has been made to increase the motivation of Spanish primary care teams by benchmarking, rating individual as well as team success. The team sets specific goals they wish to achieve jointly. Thus, the team creates shared objectives (Int-Sp8, 81). Special team funding has also been introduced as a financial incentive for high team performance; teams rewarded these funds may use them for joint activities such as advanced training. However, various interviewees maintained that this particular strategy has had little effect on the quality of co-operation in their teams (Int-Sp6, 265).

In summary: When introducing health centres in the mid-1980s, Spain’s government simultaneously focused on setting up team-based care. Since then, many health centres have worked hard on refining their concept of a primary care team. Regarding the GP-nurse collaboration, it should be noted that Spanish nurses have their own domain of responsibility (not simply regarding certain well-defined tasks). The nurses now collaborate with the GPs on terms that are more equal; nevertheless, personal conflicts can lead to professional disagreements. As a result, supporting structures for team development have been implemented. Particularly interesting is the fact that Spanish cross-professional teams have developed their own strategies to improve teamwork; for example, by shifting task responsibilities from physicians to the nurses in bottom-up processes. As indicated by our analysis, both these aspects – the increased professionalisation and autonomy of nurses and the team development – are vital factors for high-quality teamwork.

## Conclusion and discussion

Like many other countries today, Spain and Slovenia are faced with new challenges in primary care created by a changing spectrum of illness and disorders and the growing necessity to find needs-based solutions for complex, long-term health problems. As our analysis shows, both countries strive to ensure comprehensive, ongoing care and to develop new services for the patients. In order to do this, they also both have chosen to intensify team-based collaboration between GPs and nurses as the key to increasing the effectivity of care services in their health centres. Among other things, our study demonstrates that developments initiated in primary health care, and the outcomes of changes made are influenced by each country’s care history and traditions. Nevertheless, despite cultural differences, the opportunities for and quality of co-operation between GPs and nurses in Spain and Slovenian appear closely linked with the training, task responsibilities of nurses in primary care as well as status within the team. GP-nurse tandems in Spain and Slovenian were most effective, where the position of nurses in primary care had been strengthened and a new, more prominent role of nurses in the care process was accepted.

Thus, a central finding of our analysis is that traditional hierarchies between the professions hinder the possibilities of cross-professional teamwork. Structural hierarchies between the professions continue to exist not only in Slovenia and Spain, as shown here, but also in many other countries (cf. Delamaire and Lafortune, 2010; Schaeffer and Hämel, 2017). Although similar issues were discussed extensively in the 1980s (cf. Freidson, 1986; Light, 1988), they often have been overlooked in the current discussion concerning improvements of cross-professional co-operation (Paradis and Whitehead, 2015). As demonstrated, changing of the hitherto hierarchical structures of co-operation can be difficult without overcoming differences in status and the long-established power gap. The difference in status between the health professions is reflected in the still distinct educational gap between physicians and nurses (as well as other health care professions). Consequently, a key step towards changing given hierarchies entails the further professionalisation of nurses and other health professionals. The academisation of the nursing profession, for example, raises nurses’ status in the society, and thus assists their emancipation from subordinated role placement in health care. As de Geest *et al*. (2008) pointed out, education is a major driver in the development of the advanced practice nursing roles. In Spain and Slovenia, both countries have taken initial steps to integrate nursing and other health professions into higher education. Nevertheless, these professions have few opportunities in postgraduate education, and as of yet, the offered study programmes do not correspond to those in the medical fields.

In addition, our findings imply that effective change in dominance patterns and responsibility must be actively supported at the grassroots level, that is locally, in individual teams. Changes in co-operation and communication patterns, which are necessary for successful teamwork, do not occur automatically in the process of introducing teamwork. Supportive processes for change must be implemented systematically and deliberately adopted by all team members. A closer look at the local team performances in both countries gives a clearer picture on options to promote (or hinder) cross-professional teamwork:

(1) Our findings confirm the importance of *clearly defined structures* for teamwork by means of agreed definition of tasks and/or areas of responsibility. Moreover, the *mutual acknowledgement* and *understanding* of each profession’s scope of practice are a key factor in promoting cross-professional teamwork (cf. Clarin, 2007; Jaruseviciene *et al*., 2013): In both Spain and Slovenian, well-structured procedures and clear task assignment assisted the implementation of new methods. Misunderstandings and/or backslides into long-established roles, and behavioural patterns were prevented or counteracted through active and continuous communication between the participating professionals. This allowed nurses the needed space to test their new capabilities and grow into their (new) responsibilities. In Slovenia, responsibilities and tasks were defined by means of protocols established in the initial introduction of practice nurses into GPs practice. In Spain, the need for further clarification of procedures and tasks often became prevalent in the course of daily practice as nurses delved into new areas of responsibility. It would be interesting to focus further research on the appropriateness of the different procedural approaches (protocols and detailed description of tasks versus definition of areas of responsibilities). Our study indicates that particularly in health centres with little experience in cross-professional teamwork and a hitherto hierarchically oriented understanding between doctors and nurses; it could be beneficial initially to define tasks assigned to the nurses clearly, and in the process give them the needed space to develop new roles.
(2) Our results also indicate that clear definitions of tasks and responsibilities alone do not provide a sufficient basis for collaborative action in a cross-professional team. Rather, the professionals must learn how to work jointly. Particularly, in situations where unilateral profession-oriented interests dominate relationships, collaborative teamwork can become difficult. This also holds true when concerns arise in the team caused by disparate interpretations of function and task descriptions. Our study gives evidence that *shared visions of the professionals* such as patient-oriented care (cf. WHO, 2015) can provide additional guidance and motivation for collaboration. However, in the process of developing shared visions, misunderstandings can influence the discussion on the development of teamwork and the expansion of care negatively. As demonstrated above, there are risks, for example, in arguing for more task responsibility for nurses to counter a shortage of physicians or to save costs as opposed to emphasising the advantages for patients. Instead of encouraging collaborative behaviour, such arguments tend to buttress unilateral interests and short-term objectives.
(3) Finally, opportunities for cross-professional teamwork lie in the increased accessibility of expertise and skills in patient care to all team members (inter-professional expertise). Effective cross-professional teams provide *time and space for team development processes* and systematically promote them. Our study shows that, when initiating change, particularly by means of task shifting, collaborative negotiations on work procedures in the team are particularly important. The time thus made available can also be used to address possible personal conflicts between the team members, which otherwise might be ignored due to demanding daily routines. In Spain, a bottom-up design has been implemented for team’s development, the results of which look quite promising for the further development of the team’s tasks.^19^ Regular team meetings and the exchange of experiences provide opportunities not only to strengthen teamwork top-down but also to develop it bottom-up further by utilising the expertise of the participants. Interestingly enough, providing time and space for team development appears to be of greater relevance for team performance in Spain than financial incentives. In Slovenia, the full potential of team development processes has yet to be utilised. Introducing strategies to develop team development processes further will be an important task toward improving the effectivity of their cross-professional teams in the future.

Our stated aim of this paper was to take a closer look at possibilities of co-operation between GPs and nurses in primary care in Slovenia and Spain in the context of their country-specific primary care concepts and further refinement. The comparative analysis enabled us to identify differences in conceptual and procedural methods employed in the two countries; this provided us with valuable insights into various aspects of change in specific social-cultural frames. We confirmed that clearly defined structures, shared visions of the professionals and opportunities for team development are of great relevance for the effective implementation and sustainability of collaboration between general practitioners and nurses in primary care. However, in the course of the analysis, we also uncovered common challenges and typical difficulties, for example when attempting to increase responsibility, autonomy and status of nurses in the team. When developing and implementing care concepts that aim to strengthen the collaboration between physicians and nurses in the future, these problems and issues should be anticipated and confronted proactively. Further means of dealing with conflicts within the teams need to be developed and tested.

## Limitations

When appraising these research findings, it is important to take into account that the selected health centres were recommended as best-practice facilities in the two countries. Using a different sampling strategy, greater challenges and further obstacles to co-operation between physicians and nurses might have become visible. For example, in the health centres investigated here, the administration clearly supported attempts to strengthen co-operation between nurses and physicians. The management’s support and advocacy of teamwork are vital for its evolvement (CHSRF, 2006; Morgan *et al*., 2015).

It must also be noted that health centres visited in Spain were limited to only two regions, Catalonia and Basque Country. Given practical limitations of research, we chose to focus on gathering diverse perspectives from a broad spectrum of actors active in a few health centres and thus assuring a robust analysis rather than investigating a multitude of health centres. Nevertheless, our findings indicate that the assessments and concepts of ‘successful’ teamwork put forward by the GPs and nurses interviewed diverge according to their differing role definitions and expectations for their own and other professions. An in-depth analysis of these diverging perceptions could help us better understand the opportunities and obstacles of co-operation between physicians and nurses and develop viable strategies and concepts for strengthening co-operation.

Finally, it should be pointed out that the focus of this paper was on the collaboration between nurses and physicians. Many primary care concepts today, however, encompass cross-professional collaboration with a much broader spectrum of professionals. Due to the increased complexity of the patient’s needs today, the need to work closely with one another continues to increase. More research is also needed in this area.

## Footnotes

1 With reference to Thylefors *et al*. (2005: 112), we use the term ‘cross-professional teamwork’ in the broader sense to indicate ‘all situations where professionals from different disciplines are collaborating in a team. It does not say anything about how the teamwork is organised’.

2 The data used here was gathered for the research project ‘Primary Health Centres – Concepts and Practices’ (PriKon, 2015–2017; funded by the Robert Bosch Foundation, Germany), which is comparing concepts and practices of primary health centres in Slovenia, Spain, Sweden and Brazil.

3 Eight of the 10 expert interviews conducted were telephone interviews. Two experts received open-ended questionnaires containing our central questions.

4 In Slovenia, this encompassed seven expert interviews, three group discussions, three recorded presentations and seven recorded observations of the tours of the health centres. In Spain, 13 expert interviews and two group discussions were conducted. In addition, three recorded presentations and seven recorded observations during of the tours of the health centres were collected (see Supplementary material Tables A2 and A3).

5 The academisation of nursing education began in 1993 and encompassed general primary health care with an emphasis on health promotion and prevention (Albreht *et al*., 2016: 107).

6 Most private practices in Slovenia have contracts with the national health insurance, which enable them to get public funding for their services (Albreht *et al*., 2016: 111); 20–23% of the primary health care physicians and 15% of the community nurses work in such private practices (Albreht *et al*., 2016: 118, 137).

7 Registered nurses have a bachelor’s degree in nursing (Albreht *et al*., 2016: 107).

8 Roughly 11% of the contacts in specialist (secondary) outpatient care occur in these centres since medical specialists such as radiologists are employed in the larger health centres (Albreht *et al*., 2016: 122; Int-Slo9).

9 Community nurses are registered nurses with a minimum of five years practical work experience (Int-Slo5-613).

10 The health insurance in Slovenia covers two visits per year (Int-Slo5, 286).

11 The interviewees spoke English as their second language; grammar and language usage has been corrected in part for easier reading.

12 The nurses’ training focuses on eight chronic diseases: hypertension, diabetes, COPD, asthma, osteoporosis, depression, prostate cancer, cardiovascular diseases (Albreht *et al*., 2016: 121).

13 Some health centres have been set up as public-private partnerships, others are privately owned (eg, professional cooperatives) and have health care contracts with the regional health authorities (Martínez-Garcia, 2004; Ensenyat, 2007).

14 However, Spain’s modern attitude of the nursing profession does appear to have limitations: while physicians were offered the opportunity for a postgraduate specialisation in ‘family and community medicine’ as early as 1978/1979, the implementation of a similar postgraduate specialisation for nurses initially drafted in 2005 has been delayed (Dedeu *et al*., 2015: 257; Int-Sp14).

15 In total, nurses carry out close to half of all consultations done (García-Armesto *et al*., 2010).

16 For example, courses for patient groups with diabetes, courses for dealing with depression or health promotion in schools.

17 When necessary, cooperation can be extended including additional members of the (expanded) cross-professional primary health care team, for example in the process of care planning, a social worker (European Observatory on Health Care Systems 2000: 66; Int-Slo12–560).

18 A legal framework for prescribing medications was introduced in 2013 (Romero-Collado *et al*., 2014).

19 We have similar results for Brazil (see Hämel *et al*., 2017).

## Supplementary material

For supplementary material accompanying this paper visit http://dx.doi.org/10.1017/S1463423617000354. click here to view supplementary material
