# Supplementary material for: The collaboration of general practitioners and nurses in primary care: a comparative analysis of concepts and practices in Slovenia and Spain
Source: Prim Health Care Res Dev. 2017 Jun 20;18(5):492–506. doi: 10.1017/S1463423617000354 (PMC5577633; doi:10.1017/S1463423617000354)
Supplement: Supplementary file 1 [file S1463423617000354sup001.docx]

**Appendix**

**Table A.1:** Interviews with experts^1^

| **Number** | **Focus** | **Interviewees** | **Data preparation** |
| --- | --- | --- | --- |
| E1 | comparative^2^ | 1 Researcher (Primary Care) | Summary |
| E2 | comparative^2^ | 1 Researcher (General Practice) | Summary |
| E3 | Slovenia | 1 Researcher (Nursing Science) | Transcript |
| E4 | Slovenia | 1 Manager (GP)  1 Manager (Nurse) | Transcript |
| E5 | Spain | 1 Manager (Economist)  1 Manager (GP) | Transcript |
| E6 | Spain | 1 GP | Transcript |
| E7 | Spain | 1 Researcher (Epidemiology, Primary Care, Public Health) | Summary |
| E8 | Spain | 1 Researcher (Nursing Science) | Transcript |
| E9 | Spain | 1 Researcher (Nursing Science) | Written answers |
| E10 | Spain | 1 Researcher (Public Health, Primary Care, Nursing Science) | Written answers |

^1^ Telephone interview conducted before the field visits

^2^ Comparison of the countries

**Table A.2:** Slovenia – List of interviews, group discussions, presentations and recorded observations

| **Number** | **Type** | **Interviewees/Presenter (Profession)** | **Data preparation** |
| --- | --- | --- | --- |
|  | Interview | 2 Nurses | Transcript |
|  | Interview | 1 GP | Transcript |
|  | Interview | 1 Manager + Practitioner^3^ (GP)  1 Nurse | Transcript |
|  | Interview | 1 GP  1 Nurse | Transcript |
|  | Interview | 3 Nurses | Transcript |
|  | Group discussion | 1 Researcher (Nursing Science)  1 Researcher (Social Science) | Summary |
|  | Group discussion | 1 Manager + Practitioner (GP) | Summary |
|  | Presentation, Interview | 1 Nurse | Summary + Section-Transcript |
|  | Presentation | 1 Manager + Practitioner (GP)  1 Manager (GP) | Summary + Section-Transcript |
|  | Presentation,  Group discussion | 1 Manager + Practitioner (GP)  2 Manager + Practitioner (Nurse) | Summary + Section-Transcript |
|  | Presentation, Interview | 1 Manager + Practitioner (GP)  1 Manager + Practitioner (Nurse)  1 Manager (Economist) | Transcript |
|  | Recorded observation | 1 Manager + Practitioner (GP)  1 Manager (GP) | Summary |
|  | Recorded observation | 1 Manager + Practitioner (GP) | Summary |
|  | Recorded observation | 1 Nurse | Summary |
|  | Recorded observation | 1 Manager (Nurse) | Summary |
|  | Recorded observation | 1 Nurse  1 Manager (Nurse) | Summary |
|  | Recorded observation | 2 GPs  1 Manager (GP) | Summary |
|  | Recorded observation | 1 Nurse | Summary |

^3^ The label ‘Practitioner’ stands for persons involved in management as well as in patient care. These interviews addressed their management/organisational concepts as well as their own practical care experience.

**Table A.3:** Spain – List of interviews, group discussions, presentations and recorded observations

| **Number** | **Type** | **Interviewees/Presenter (Profession)** | **Data preparation** |
| --- | --- | --- | --- |
|  | Interview | 1 Nurse | Transcript |
|  | Interview | 1 Manager (GP) | Transcript + summary |
|  | Interview | 1 Manager (Economist) | Transcript |
|  | Interview | 1 Administrative Staff | Summary |
|  | Interview | 1 Manager (Economist) | Transcript |
|  | Interview | 1 Manager + Practitioner^4^ (GP)  1 GP  1 Nurse | Transcript |
|  | Interview | 2 GPs | Transcript |
|  | Interview | 2 Nurses | Transcript |
|  | Interview | 1 Social worker | Summary |
|  | Interview | 1 GP | Transcript |
|  | Interview | 1 Manager + Practitioner (GP)  1 GP | Transcript |
|  | Interview | 2 Nurses | Transcript |
|  | Group discussion | 1 GP  2 Nurses | Transcript |
|  | Group discussion | 2 Researcher (Nursing Science) | Summary |
|  | Presentation | 1 Manager (Economist) | Summary |
|  | Presentation | 2 Managers (Nurses)  1 Nurse | Summary |
|  | Presentation,  Interview | 3 Nurses | Transcript |
|  | Recorded observation | 1 Manager (Economist) | Summary |
|  | Recorded observation | 1 Manager (Economist) | Summary |
|  | Recorded observation | 1 Manager (Economist) | Summary |
|  | Recorded observation | 1 Manager + Practitioner (GP)  1 GP | Summary |
|  | Recorded observation | 1 Manager + Practitioner (GP)  1 GP | Summary |
|  | Recorded observation | 1 GP | Transcript |
|  | Recorded observation | 1 Nurse  1 Researcher (Nursing Science) | Summary |

^4^ The label ‘Practitioner’ stands for persons involved in management as well as in patient care. These interviews addressed their management/organisational concepts as well as their own practical care experience.
